# *Mycobacterium caprae* Infection in Livestock and Wildlife, Spain

**DOI:** 10.3201/eid1703.100618

**Published:** 2011-03

**Authors:** Sabrina Rodríguez, Javier Bezos, Beatriz Romero, Lucía de Juan, Julio Álvarez, Elena Castellanos, Nuria Moya, Francisco Lozano, M. Tariq Javed, José L. Sáez-Llorente, Ernesto Liébana, Ana Mateos, Lucas Domínguez, Alicia Aranaz, The Spanish Network on Surveillance, Monitoring of Animal Tuberculosis

**Affiliations:** Author affiliations: Universidad Complutense de Madrid, Madrid, Spain (S. Rodríguez, J. Bezos, B. Romero, L. de Juan, J. Álvarez, E. Castellanos, N. Moya, F. Lozano, A. Mateos, L. Domínguez, A. Aranaz);; University of Agriculture, Faisalabad, Pakistan (M.T. Javed);; Ministerio de Medio Ambiente, y Medio Rural y Marino, Madrid, (J.L. Sáez-Llorente);; European Food Safety Authority, Parma, Italy (E. Liébana)

**Keywords:** Bacteria, zoonoses, Mycobacterium caprae, tuberculosis and other mycobacteria, epidemiology, molecular characterization, livestock, goats, spoligotyping, dispatch

## Abstract

*Mycobacterium caprae* is a pathogen that can infect animals and humans. To better understand the epidemiology of *M. caprae*, we spoligotyped 791 animal isolates. Results suggest infection is widespread in Spain, affecting 6 domestic and wild animal species. The epidemiology is driven by infections in caprids, although the organism has emerged in cattle.

*Mycobacterium caprae* is a cluster within the *M. tuberculosis* complex (Technical Appendix). This pathogen has been recognized mainly in central Europe, where it has been occasionally isolated from tuberculous lesions from cattle (*1**–**5*), pigs (*4*), red deer (*Cervus elaphus*) (*4**,**5*), and wild boars (*Sus scrofa*) (*3*). Its isolation from humans has also been described (*3**,**6*); often, a contact with livestock has been suggested as a likely means of transmission (*5*). To our knowledge, this pathogen has never been isolated outside continental Europe, except from a European patient in Australia (*7*) and a cow in Algeria (*8*).

The combination of disease tracing and molecular typing is needed to understand the epidemiology of tuberculosis. This report describes the molecular epidemiology of *M. caprae* infection in Spain compared with other countries. We characterized *M. caprae* isolates from goats and other domestic and wild animals by spoligotyping (*9*). The relative contribution of each animal and its role in animal tuberculosis are discussed.

## The Study

**Figure 1:**
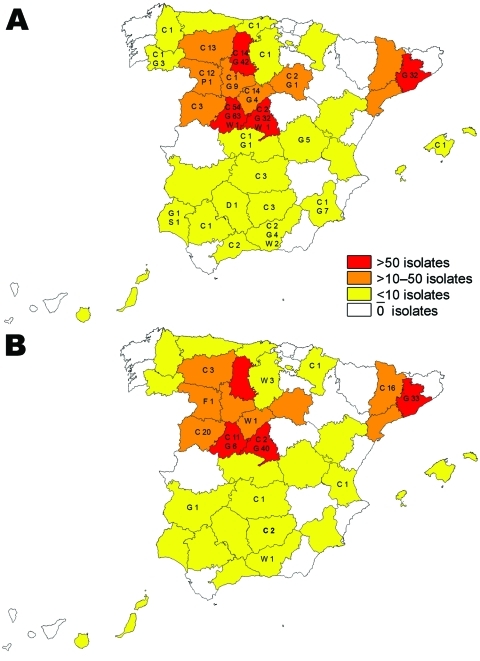
Map of Spain showing the distribution of the 2 most frequent *Mycobacterium caprae* spoligotypes and affected animals: C, cattle; D, red deer; F, fox; G, goats; S, sheep; P, pigs; WB, wild boar. A) Spoligotype SB0157. B) Spoligotype SB0416.

This study included 791 *M. caprae* isolates from domestic goats (*Capra aegagrus hircus*, n = 542), sheep (*Ovis aries*, n = 2), cattle (*Bos taurus*, n = 229), domestic pigs (*Sus scrofa domestica*, n = 2), wild boars (*Sus scrofa*, n = 14), red deer (*Cervus elaphus*, n = 1), and a fox (*Vulpes vulpes*, n = 1). The samples originated from skin test–positive animals identified within the national or regional eradication programs, from abattoir surveillance, and from postmortem inspections of wildlife, and were collected from 1992 through June 2009 in different geographic areas in Spain (Figure 1). Spoligotyping was performed as described (*9*), and authoritative names for spoligotype patterns were obtained from the *Mycobacterium bovis* Spoligotype Database (www.mbovis.org).

Further authentication was achieved by detection of RD4 in the isolates with a 3-primer PCR in a panel of 63 unrelated isolates that included all spoligotyping patterns and animal species. Of the selected isolates, 62 showed the 545-bp product, indicating that they harbor RD4. One isolate from a cow of Eastern European origin repeatedly showed a 340-bp band, and its sequencing could not confirm presence or absence of RD4. For detection of specific *M. caprae* gene polymorphisms, 1 isolate from every spoligotyping pattern was studied. Additional identification was determined by sequencing of the pyrazinamidase A gene, which demonstrated a C at nt 169 that results in the functional wild-type pyrazinamidase A gene, and of the gyrase B gene that showed the G at nt 1311 and a C at position 1410 (Technical Appendix).

The isolates, which originated from 195 single cases or outbreaks (Table 1), clustered into 15 patterns, which share the features previously described for the species (absence of spacers 1, 3–16, 28, and 39–43). Notably, the Iberian spoligotype cluster lacks spacers 30–33, whereas most *M. caprae* isolates from central Europe belong to spoligotypes that harbor these spacers. The 3 isolates of profiles SB0418 and SB1619 that presented spacers 30–33 originated from cattle imported from southeastern Europe. The 2 predominant spoligotypes, SB0157 and SB0416, were found to be responsible for 60% and 22%, respectively, of the cases and infected different animal species in distant areas, whereas 7 patterns were unique to a single case or outbreak. We calculated the index of discrimination (D) described by Hunter and Gaston (*10*) using the website of the University of the Basque Country (www.insilico.ehu.es). The result, D = 0.584, is notably lower compared with a parallel research of 252 patterns from 6,215 *M. bovis* isolates (D = 0.87) (*11*).

**Table 1:** Spoligotyping results of 791 *Mycobacterium caprae* isolates and their distribution within different animal species, Spain, 1992–2009*

Ref	Spoligotyping pattern†	No. animals (no. outbreaks)						
Goats	Sheep	Cattle	Pigs	Wild boar	Red deer	Fox
SB0157	□■□□□□□□□□□□□□□□■■■■■□□■■■■□■□□□□■■■■■□□□□□	204 (44)	1	133 (67)	1	4 (4)	1	
SB0415	□■□□□□□□□□□□□□□□■■□■■■■■■■■□■□□□□■■■■■□□□□□	37 (4)	1	8 (4)		4 (3)		
SB0416	□■□□□□□□□□□□□□□□■■■■■■■■■■■□■□□□□■■■■■□□□□□	80 (18)		58 (21)		5 (3)		1
SB0866	□■□□□□□□□□□□□□□□■■■■■■■■□□□□□□□□□□□□□□□□□□□	1		1	1			
SB0973	□□□□□□□□□□□□□□□□■■■■■□□■■■■□■□□□□□■□■■□□□□□	1						
SB1077	□■□□□□□□□□□□□□□□■■■■■■■■■□□□■□□□□■■■■■□□□□□			17 (4)		1		
SB1078	□■□□□□□□□□□□□□□□■■■■□□□■■■■□■□□□□■■■■■□□□□□	1		1				
SB1079	□□□□□□□□□□□□□□□□■■■■□□□■■■■□■□□□□□■□■■□□□□□	1						
SB1080	□■□□□□□□□□□□□□□□■■■■■■■■■■□□■□□□□■■■■■□□□□□	2 (1)						
SB1081	□■□□□□□□□□□□□□□□■■■■■□□■□□□□□□□□□□□□□□□□□□□	1		8 (4)				
SB1084	□■□□□□□□□□□□□□□□■■□■■■■□■■■□■□□□□■■■■■□□□□□	211 (1)						
SB1889	□■□□□□□□□□□□□□□□■■□■■■■□□□□□□□□□□□□□□□□□□□□	2 (1)						
SB1872	□□□□□□□□□□□□□□□□□□■■■□□■■■■□■□□□□■■■■■□□□□□	1						
SB0418	□■□□□□□□□□□□□□□□■■■■■■■■■■■□■■■■■■■■■■□□□□□			2 (2)				
SB1690	□■□□□□□□□□□□□□□□■■■■■■□■■■■□■■■■■■■■■■□□□□□			1				
Total		542 (75)	2 (2)	229 (105)	2 (2)	14 (11)	1	1

*Ref, reference. Numbering according to www.Mbovis.org. **†**■, presence of spacer ; □, absence of spacer.

Additionally, variable number tandem repeat typing by using loci ETR-A, ETR-B, ETR-D, QUB11a, QUB11b, QUB3232, ETR-E, and MIRU26 ( Technical Appendix) was performed as described by Frothingham and Meeker-O’Connell (*12*) on a selection of 20 isolates (Table 2). The isolates originated from 10 properties (6 goat herds and 4 cattle farms), each with 2 different spoligotypes detected at a time. At 5 farms, the loss of spacers 25–27, 29, and 34–38, which can be explained by a single deletion event, had caused a change of the spoligotype pattern. This loss changed SB0157 to SB1081 and SB1084 to SB1889, while the variable number tandem repeat profiles within the same farm remained identical.

**Table 2:** Variable number tandem repeat analysis of isolates from 10 farms that presented mixed *Mycobacterium caprae* infection (different spoligotype patterns), Spain, 1992–2009*

Farm	Animal	Spoligotype	No. alleles at locus							
			ETR-A	ETR-B	ETR-D	QUB3232	QUB 11a	QUB 11b	MIRU 26	MIRU 31
1	Goat	SB0416	4	4	4	8	7	2	5	2
		SB0866	5	3	3	8	7	4	2	4
2	Goat	SB0416	4	3	4	8	7	2	4	2
		SB0157	4	3	4	8	7	2	4	2
3	Goat	SB0416	4	5	5	7	6	4	5	5
		SB0415	5	1	3	8	7	3	5	5
4	Cattle	SB0157	3	3	4	8	7	2	5	2
		SB1081	3	3	4	8	7	2	5	2
5	Cattle	SB0157	4	3	4	3	7	2	5	2
		SB1081	4	3	4	3	7	2	5	2
6	Goat	SB0157	4	3	4	8	7	2	5	2
		SB1078	4	3	4	8	7	2	5	2
7	Goat	SB1084	5	1	3	9	5†	3	5	4
		SB1889	5	1	3	9	5†	3	5	4
8	Cattle	SB0157	4	3	4	8	7	2	5	2
		SB1081	4	3	4	8	7	2	5	2
9	Cattle	SB0416	5	3	3	8	6	4	2	3
		SB0157	4	3	4	8	7	2	5	2
10	Goat	SB0973	4	3	–	–	–	–	–	–
		SB0157	4	3	4	9	–	2	5	–

*–, no amplification. †Gel band of ≈1,800 bp. Sequencing showed that insertion sequence IS*6110* is inserted within the third repetition of QUB11a.

The routine application of molecular diagnosis and typing techniques in clinical laboratories has enabled its real role as a pathogen for several species to be recognized. In Spain, *M. caprae* represents 7.4% of all *M. tuberculosis* complex isolates from domestic and wild animals. Seventy-five of the 197 outbreaks (38.1%) involved goats (Table 1). This species showed the highest diversity among *M. caprae* with 12 patterns identified, 6 of them exclusive to caprine herds. The association of *M. caprae* with goats in Spain may be due to 2 reasons. First, the microorganism seems to be highly pathogenic for the goats in Spain, based on the disseminated tuberculous lesions that it produces and its fast transmission within a herd. Second, caprine herds have not been included in the national eradication campaign (except when coexisting with cattle or as part of some regional programs). Therefore, *M. caprae* infection can spread easily through animal movements, such as purchase for replacement or genetic improvement.

**Figure 2:**
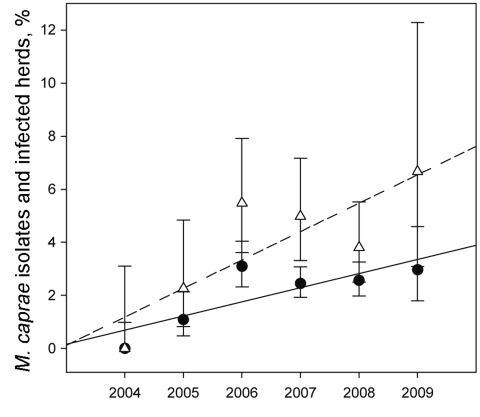
Proportion and regression lines of *Mycobacterium caprae* isolates (black dots, continuous line) and *M. caprae*–infected herds (white triangles, dashed lines) of the total number of *M. tuberculosis* complex isolates and *M. tuberculosis* complex–infected herds identified in cattle during 2004–2009. Error bars indicate 95% confidence intervals.

The emergence of this pathogen in cattle has been observed. Cattle were involved in 106 outbreaks (53.3%) during the study period. Since 2004, cattle from 2,218 herds identified in the eradication program have been inspected by bacteriology. The number of cattle properties infected with *M. caprae* represented 0.85%–6.67% of the total number of herds diagnosed with bovine tuberculosis. Temporal trend of *M. caprae* isolates cultured over time was assessed by using the software WINPEPI 9.4 (*13*). The proportion of *M. caprae* isolated from bovine samples has increased consistently during 2004–2009, showing a significant positive trend (p = 0.009, by Mantel trend test) (Figure 2). We observed more *M. caprae* infections in cattle in regions with a high goat density. However, an analysis of the type of farm production shows that 86.7% of *M. caprae*–infected cattle have been raised in farms without any contact with small ruminants. This fact indicates recirculation of the pathogen within and between cattle herds. In countries that are virtually free of animal tuberculosis such as Germany, Austria, and the Czech Republic, a large number of cases in cattle and red deer are caused by *M. caprae*.

Identification of isolates from human patients has shown *M. caprae* as a human pathogen (*3**,**6**,**14*). A recent study suggests that *M. caprae* causes 0.3% of the cases of human tuberculosis in Spain, with SB0157 also being the most dominant spoligotype (*14*). The role of the pathogen as a public health risk is highlighted by lesions that can also be found in the mammary glands of infected goats; thus, consumption of unpasteurized dairy products remains a concern (*15*).

## Conclusions

Compelling evidence indicates that *M. caprae* poses a serious health risk not only for goats, but also for other domestic and wild animal species and humans. Our results indicate that *M. caprae* infection is widespread in Spain and that the epidemiology is driven by caprine infections. Considering the role of *M. caprae* in animal tuberculosis, relevant legislation should be considered to address the infection as was done for *M. bovis*.

## Supplementary Material

Technical Appendix Specific Characteristics.
