# Supplementary material for: Mycobacterium caprae Infection in Livestock and Wildlife, Spain
Source: Emerg Infect Dis. 2011 Mar;17(3):532–5. doi: 10.3201/eid1703.100618 (PMC3165998; doi:10.3201/eid1703.100618)
Supplement: Technical Appendix — Specific Characteristics. [file 10-0618-Techapp.pdf]

Article DOI: 10.3201/eid1703.100618

Mycobacterium caprae Infection in Livestock and Wildlife, Spain

Technical Appendix

Specific Characteristics

Mycobacterium caprae (*1*), formerly known as *M. tuberculosis* subsp. *caprae* (*2*), and *M. bovis* subsp. *caprae* (*3*) forms a genetically distinct cluster within the *M. tuberculosis* complex. The main features differentiating these isolates from the other members are a special combination of polymorphisms at pyrazinamidase (*pncA*), catalase (*katG*), and subunits A and B of the gyrase (*gyrA* and *gyrβ*) genes (*4,5*); the pattern of regions of difference (presence of RD4 and absence of RD5 to 10) (*6–8*); and specific patterns obtained by direct variable repeat spacer oligonucleotide typing technique (spoligotyping); and restriction fragment length polymorphism associated with IS*6110*, polymorphic GC-rich sequences, and direct repeat elements (*9,10*).

Bacteriology

Tissue samples consisted usually of retropharyngeal, mediastinal, bronchial, and mesenteric lymph nodes, lung and liver. All samples were maintained at –20°C until culture. Samples from each animal were pooled, homogenized with sterile distilled water, decontaminated with 0.35% hexadecylpyridinium chloride for 30 min (*11*), centrifuged at 1,068 × *g* for 30 min, and cultured on Coletsos and 0.2% (w/v) pyruvate-enriched Löwenstein-Jensen media (bioMérieux España and Biomedics, Madrid, Spain) at 37°C for 3 mo. The isolates were identified as members of the *M. tuberculosis* complex by PCR amplification of *Mycobacterium* genus–specific 16S rRNA fragment (*12*) and MPB70 sequences (*13*) (primers used in the study are listed in the Table). All PCRs were performed on heat-killed cell suspensions.

Spoligotyping and Data Analysis

The spacer oligonucleotide typing (spoligotyping) method was performed as described by Kamerbeek et al. (*14*). The biotin-labelled amplified product was detected by hybridization onto a spoligotyping membrane (Isogen Bioscience BV, Maarssen, the Netherlands). Hybridized

product was detected with the streptavidin-peroxidase conjugate (Boehringer, Mannheim, Germany) and the electrochemical luminescence system (Amersham, Little Chalfont, UK) by exposing the radiograph film to the membrane. Purified sterile water and a clinical isolate of *M. tuberculosis* and *M. bovis* were included as controls in every batch of tests.

The spoligotyping results were enlisted in a Microsoft Office Access (Microsoft, Redmond, WA, USA) database along with the epidemiologic data (isolation date, animal species and geographical origin). The index of discrimination (D) described by Hunter and Gaston (*15*) was calculated to determine the discriminatory power of the spoligotyping at a national level. We used the website of the University of the Basque Country (www.insilico.ehu.es), filling in the number of unrelated strains for each spoligotype. For this purpose we only counted 1 spoligotype when isolates of the same herd or a precise geographical area shared identical patterns.

Detection of RD4 and Gene Polymorphisms

We used the 3-primer PCR described by Mostowy et al. (*16*). Purified sterile water and a clinical isolate of *M. bovis* were included as controls. The presence (545-bp gel band) or absence (210-bp gel band) of RD4 was detected by agarose gel electrophoresis.

The complete *pncA* gene (*17*) and a part of the *gyrB* (*18*) containing the expected polymorphism for *M. caprae* were amplified. The products were purified with the Qiaquick PCR Purification kit (QIAGEN GmbH, Hilden, Germany) and sequenced with the DyeDeoxy (dRhodamine) Terminator Cycle Sequencing kit in an automatic ABI Prism 373 DNA sequencer (Applied Biosystems, C.I.B. Sequencing Facilities, Madrid, Spain). The sequences generated were aligned with published mycobacterial sequences from the GenBank database (www.ncbi.nlm.nih.gov/GenBank, accession nos. U59967 [*17*] and L27512 [*18*]). Sequencing of the *pncA* demonstrated a C at nucleotide 169, a common characteristic for *M. tuberculosis*, *M. africanum, M. microti,* and *M. caprae* that results in the functional wild-type *pncA* (*17*). The *gyrβ* gene sequence polymorphisms analysis detected, as well the characteristic profile for *M. caprae* that consists of a G at nucleotide 1311 and a C at position 1410, are common to caprine strains and the other members of the complex, except *M. bovis* (*5*).

Variable Number Tandem Repeat Analysis

The PCR for each locus was carried out by using the HotStar Taq DNA polymerase kit (QIAGEN) in a Bio-Rad (Hercules, CA, USA) MyCycler Thermal Cycler. Genomic DNA from

M. bovis BCG Danish was used as a positive control, reaction mixtures lacking mycobacterial DNA were used as a negative control. The number of tandem repeats (alleles) was determined by estimating the amplicon size of the PCR product by electrophoresis on 2.5% agarose gel at 45V for 3 h with a 100-bp ladder (Biotools, B&M Labs, Madrid, Spain).

Members of the Spanish Network on Surveillance and Monitoring of Animal Tuberculosis: F. Garrido (Laboratorio Central de Sanidad Animal de Santa Fé, Granada, Ministerio de Medio Ambiente, Rural y Marino), staff of Government and Regional and Research Laboratories of Autonomous Communities (C. Fornell, J.M. Gómez, A. Jiménez, I. Muñoz, J.A. Téllez, E.J. Villalba [Andalucía], N. Abacens, I. Belanche, J. Gracia, S. Izquierdo, J.M. Malo [Aragón], M.F. Copano, E. Fernández, I. Merediz [Asturias], P. Peláez, C. Pieltain, V. Vigo [Canarias], C. Fernández, F.M. Fernández, M.G. Gradillas, M. Gutiérrez, E. Sola [Cantabria], V. Alcaide, J. Alia, J. Alonso, M.R. Bermúdez, C. Fernández, P. García, E. Grande, F. Plaza, M.L. Rando, C. Rojas, A. Sánchez, J.A. Viñuelas [Castilla La Mancha], J.A. Anguiano, I. Burón, J. Cermeño, C. Domínguez, F. Fernández, A. Grau, S. Marques, O. Martín, C. Martínez, O. Mínguez, F. Moreno, F. Reviriego, I. Romero [Castilla y León], J. Gou [Cataluña], J.R. Puy [Euskadi], E. Dorado, C. Sanz [Extremadura], C. Calvo, D. Fernández, J.E. Mourelo [Galicia], C. Aguilo, M.J. Portau, C. Vidal [Islas Baleares], J.M. Cámara, J. Carpintero, C. Delso, R. Díaz, E. Fernández, C. Fernández-Zapata, M. García, E. Pages, J.J. Urquía [Madrid], J. Pastor, C. Rivas [Murcia], J, Eguiluz, F. Eslava, C. Fernández [Navarra], F.J. Puértolas, J.F. Soldevilla [La Rioja], C. Caballero, M. Lázaro [Valencia]); A. Jacoste, M. Moreno (Patrimonio Nacional); academic and research members from Faculties of Veterinary Sciences (S. Lavin, G. Mentaberre [Universidad Autónoma de Barcelona], A. Perea [Universidad de Córdoba], A. García, J. Hermoso de Mendoza, A. Parra, [Universidad de Extremadura], E.F. Rodríguez-Ferri, O. González-Llamazares [Universidad de León], J. Blanco, M. Castaño, A.A. Díez-Guerrier, J.V. González, F. Mazzucchelli, C. Novoa, X. Pickering, M. Pizarro, G. Santurde, I. Simarro [Universidad Complutense de Madrid], A. Contreras, J. Sánchez [Universidad de Murcia], A. Fernández, O. Quesada [Universidad de Las Palmas de Gran Canaria], M.V. Latre [Universidad de Zaragoza]); colleagues from research centers on animal health (M. Domingo, B. Pérez, S. López, D. Vidal [Centre de Recerca en Sanitat Animal], J. Garrido, R. Juste [Instituto Vasco de Investigación y Desarrollo Agrario], M. Galka, C. Sánchez, [P.N. Doñana], J. de la Fuente, C. Gortázar, J. Vicente [Instituto de Investigación en Recursos Cinegéticos-Consejo Superior de Investigaciones Cientificas], A. Espí, J.M. Prieto and [Servicio Regional de Investigación y Desarrollo Agrario, Asturias], I. Carpio [Unión de Criadores del Toro de Lidia]; veterinary inspectors at abattoirs (A.J. Domínguez, M. Fernández, J.M. Rubio [Ciudad Real], M. García, J. Guedeja, F. Osuna, J.L. del Pozo [Madrid]); M.D. E. Gómez-Mampaso [H. Ramón y Cajal, Madrid] and R. Borrás [Facultad de Medicina, Valencia]); and veterinary practitioners (P. Díez de Tejada, J.M. Fernández [A.D.S. Cabra del Guadarrama, Madrid], C. Gil, F. Moneo-López, I. Larrauri [Albacete], J. Cermeño, D. Martín [Badajoz], J.L. García [Burgos], A. Rodríguez, E. Sainz [Cáceres], P.J. Mora [Ciudad Real], J.M. Amigo, N. Castro, V. Collado, J.L. Cumbreño, J.M. Finat, M.P. Herranz, E. Legaz, L.M. Portas, J. Rodríguez, L. Sánchez, J.M. Sebastián, T. Yuste [Madrid], A. Santos (Toledo), J. Fonbellida [Zamora], and J. Rodríguez [Laboratorios Syva]).

References

1. Aranaz A, Cousins D, Mateos A, Domínguez L. Elevation of *Mycobacterium tuberculosis* subsp. *caprae* Aranaz et al. 1999 to species rank as *Mycobacterium caprae* comb. nov., sp. nov. Int J Syst Evol Microbiol. 2003;53:1785–9. PubMed DOI: 10.1099/ijs.0.02532-0

2. Aranaz A, Liébana E, Gómez-Mampaso E, Galán JC, Cousins D, Ortega A, et al. *Mycobacterium tuberculosis subsp. caprae subsp. nov.*: a taxonomic study of a new member of the *Mycobacterium tuberculosis* complex isolated from goats in Spain. Int J Syst Bacteriol. 1999;49:1263–73. PubMed DOI: 10.1099/00207713-49-3-1263

3. Niemann S, Richter E, Rüsch-Gerdes S. Biochemical and genetic evidence for the transfer of *Mycobacterium tuberculosis* subsp. *caprae* Aranaz et al. 1999 to the species *Mycobacterium bovis* Karlson and Lessel 1970 (approved lists 1980) as *Mycobacterium bovis* subsp. *caprae* comb. nov. Int J Syst Evol Microbiol. 2002;52:433–6. PubMed

4. Espinosa de los Monteros LE, Galán JC, Gutiérrez M, Samper S, García Marin JF, Martin C, et al. Allele-specific PCR method based on *pncA* and *oxyR* sequences for distinguishing *Mycobacterium bovis* from *Mycobacterium tuberculosis*: intraspecific *M. bovis pncA* sequence polymorphism. J Clin Microbiol. 1998;36:239–42. PubMed

5. Niemann S, Harmsen D, Rüsch-Gerdes S, Richter E. Differentiation of clinical *Mycobacterium tuberculosis* complex isolates by *gyrB* DNA sequence polymorphism analysis. J Clin Microbiol. 2000;38:3231–4. PubMed

6. Mostowy S, Inwald J, Gordon S, Martín C, Warren R, Kremer K, et al. Revisiting the evolution of *Mycobacterium bovis.* J Bacteriol. 2005;187:6386–95. PubMed DOI: 10.1128/JB.187.18.6386-6395.2005

7. Huard RC, Fabre M, de Haas P, Lazzarini LC, van Soolingen D, Cousins D, et al. Novel genetic polymorphisms that further delineate the phylogeny of the *Mycobacterium tuberculosis* complex. J Bacteriol. 2006;188:4271–87. PubMed DOI: 10.1128/JB.01783-05

8. Brosch R, Gordon SV, Marmiesse M, Brodin P, Buchrieser C, Eiglmeier K, et al. A new evolutionary scenario for the *Mycobacterium tuberculosis* complex. Proc Natl Acad Sci U S A. 2002;99:3684–9. PubMed DOI: 10.1073/pnas.052548299

9. Aranaz A, Liébana E, Mateos A, Domínguez L, Cousins D. Restriction fragment length polymorphism and spacer oligonucleotide typing: a comparative analysis of fingerprinting strategies for

Mycobacterium bovis. Vet Microbiol. 1998;61:311–24. PubMed DOI: 10.1016/S0378-1135(98)00192-8

10. Gutiérrez M, Samper S, Gavigan JA, García Marín JF, Martín C. Differentiation by molecular typing of *Mycobacterium bovis* strains causing tuberculosis in cattle and goats. J Clin Microbiol. 1995;33:2953–6. PubMed

11. Corner LA, Trajstman AC. An evaluation of 1-hexadecylpyridinium chloride as a decontaminant in the primary isolation of *Mycobacterium bovis* from bovine lesions. Vet Microbiol. 1988;18:127–34. PubMed DOI: 10.1016/0378-1135(88)90058-2

12. Böddinghaus B, Rogall T, Flohr T, Blöcker H, Böttger EC. Detection and identification of mycobacteria by amplification of rRNA. J Clin Microbiol. 1990;28:1751–9. PubMed

13. Wilton S, Cousins D. Detection and identification of multiple mycobacterial pathogens by DNA amplification in a single tube. PCR Methods Appl. 1992;1:269–73. PubMed

14. Kamerbeek J, Schouls L, Kolk A, van Agterveld M, van Soolingen D, Kuijper S, et al. Simultaneous detection and strain differentiation of *Mycobacterium tuberculosis* for diagnosis and epidemiology. J Clin Microbiol. 1997;35:907–14. PubMed

15. Hunter PR, Gaston MA. Numerical index of the discriminatory ability of typing systems: an application of Simpson's index of diversity. J Clin Microbiol. 1988;26:2465–6. PubMed

16. Mostowy S, Cousins D, Brinkman J, Aranaz A, Behr MA. Genomic deletions suggest a phylogeny for the *Mycobacterium tuberculosis* complex. J Infect Dis. 2002;186:74–80. PubMed DOI: 10.1086/341068

17. Scorpio A, Zhang Y. Mutations in *pncA*, a gene encoding pyrazinamidase/nicotinamidase, cause resistance to the antituberculous drug pyrazinamide in tubercle bacillus. Nat Med. 1996;2:662–7. PubMed DOI: 10.1038/nm0696-662

18. Kasai H, Ezaki T, Harayama S. Differentiation of phylogenetically related slowly growing mycobacteria by their *gyrB* sequences. J Clin Microbiol. 2000;38:301–8. PubMed

19. Allix C, Walravens K, Saegerman C, Godfroid J, Supply P, Fauville-Dufaux M. Evaluation of the epidemiological relevance of variable-number tandem-repeat genotyping of *Mycobacterium bovis* and comparison of the method with *IS*6110 restriction fragment length polymorphism analysis and spoligotyping. J Clin Microbiol. 2006;44:1951–62. PubMed DOI: 10.1128/JCM.01775-05

20. Frothingham R, Meeker-O'Connell WA. Genetic diversity in the *Mycobacterium tuberculosis* complex based on variable numbers of tandem DNA repeats. Microbiology. 1998;144:1189–96. PubMed DOI: 10.1099/00221287-144-5-1189

21. Supply P. Protocol and Guidelines for Multilocus Variable Number Tandem Repeat Genotyping of *M. bovis* VENoMYC (Veterinary Network of Laboratories Researching into Improved Diagnosis and Epidemiology of Mycobacterial Diseases) WP7 Workshop, October 19-22 2006, Toledo, Spain, pp.15-16. WP7 Workshop VENoMYC Coordination Action EU SSPE-CT-2004-501903. 2006

22. Supply P, Lesjean S, Savine E, Kremer K, van Soolingen D, Locht C. Automated high-throughput genotyping for study of global epidemiology of *Mycobacterium tuberculosis* based on mycobacterial interspersed repetitive units. J Clin Microbiol. 2001;39:3563–71. PubMed DOI: 10.1128/JCM.39.10.3563-3571.2001

23. O’Brien R, Danilowicz BS, Bailey L, Flynn O, Costello E, O’Grady D, et al. Characterization of the *Mycobacterium bovis* restriction fragment length polymorphism DNA probe pUCD and performance comparison with standard methods. J Clin Microbiol. 2000;38:3362–9. PubMed

24. Skuce RA, McCorry TP, McCarroll JF, Roring SM, Scott AN, Brittain D, et al. Discrimination of *Mycobacterium tuberculosis* complex bacteria using novel VNTR-PCR targets. Microbiology. 2002;148:519–28. PubMed

Table. List of primers used in a study of *Mycobacterium caprae* infection in livestock and wildlife, Spain*

Target†	Primer	Sequence, 5′ → 3′	Product, bp	Reference
16S rRNA	MYCGEN-F<br>MYCGEN-R	AGAGTTTGATCCTGGCTCAG<br>TGCACACAGGCCACAAGGGA	1,030	*(12)*
MPB70	TB1-F<br>TB1-R	GAACAATCCGGAGTTGACAA<br>AGCACGCTGTCAATCATGTA	372	*(13)*
DR spoligotyping	DR-a<br>DR-b	GGTTTTGGGTCTGACGAC<br>CCGAGAGGGGACGGAAAC	ladder	*(14)*
RD4	RD4-L<br>RD4-R<br>RD4-wtR	GAACGCGACGACCTCATATTCC<br>CTAAGATATCCGGTACGCCCGC<br>CTGTGGCTATGGGGCTCTAC	545/210 (presence/ absence)	*(6,16)*
pncA	pncATB-1<br>pncATB-2	ATGCGGGCGTTGATCATCGT<br>TCAGGAGCTGCAAACCAACTC	574	*(4,17)*
gyrB	MTUBf<br>MTUBr	TCGGACGCGTATGCGATATC<br>ACATACAGTTCGGACTTGCG	1,020	*(5,18)*
VNTR2165<br>(ETR-A)	ETRA-F<br>ETRA-R	AAATCGGTCCCATCACCTTCTTAT<br>CGAAGCCTGGGGTGCCCGCGATTT	†	*(19)*
VNTR2461<br>(ETR-B)	ETRB-F<br>ETRB-R	GCGAACACCAGGACAGCATCATG<br>GGCATGCCGGTGATCGAGTGG	†	*(20)*
VNTR580<br>(ETR-D, MIRU 4)	ETRD-F<br>ETRD-R	GCGCGAGAGCCCGAACTGC<br>GCGCAGCAGAAACGCCAGC	†	*(19,21)*
VNTR3192<br>(ETR E, MIRU 31)	MIRU31-F<br>MIRU31-R	ACTGATTGGCTTCATACGGCTTTA<br>GTGCCGACGTGGTCTTGAT	†	*(22)*
VNTR2996<br>(MIRU 26)	MIRU26-F<br>MIRU26-R	TAGGTCTACCGTCGAAATCTGTGAC<br>CATAGGCGACCAGGCGAATAG	†	*(21)*
VNTR2163a<br>(QUB11a)	QUB11a-F<br>QUB11a-R	CCCATCCCGCTTAGCACATTCGTA<br>TTCAGGGGGGATCCGGGA	†	*(23,24)*
VNTR2163b<br>(QUB11b)	QUB11b-F<br>QUB11b-R	CGTAAGGGGGATGCGGGAAATAGG<br>CGAAGTGAATGGTGGCAT	†	*(23,24)*
VNTR3232<br>(QUB3232)	3232-F<br>3232-R	CGGCGATGGTGCCGCCATG<br>CTTGGTGAAGGCCCCGATG	†	*(21)*

*VNTR, variable number tandem repeat; MIRU, mycobacterial interspersed repetitive unit.
†According to respective allele calling tables.
